# Stable transformation mediated by *Agrobacterium tumefaciens* in Jonquil is better than *Agrobacterium rhizogenes*

**DOI:** 10.3389/fpls.2025.1594197

**Published:** 2025-06-26

**Authors:** Wenjie Yu, Haoran Tian, Yifan Chen, Shanhua Lyu, Yinglun Fan

**Affiliations:** College of Agriculture and Biology, Liaocheng University, Liaocheng, China

**Keywords:** Jonquil (*Kalanchoe blossfeldiana*), leaf propagation, *Agrobacterium tumefaciens*, *Agrobacterium rhizogenes*, genetic transformation, leaf-cutting-transformation

## Abstract

*Agrobacterium tumefaciens-*mediated genetic transformation has become an important method to study gene function and create new germplasm in plants. The classic genetic transformation process is time-consuming and laborious. In nature, some plants can propagate through detached leaves, which shows that the detached leaves can complete the dedifferentiation of leaf cell and bud differentiation through the intracellular hormone regulation of detached leaves without external hormones. Here, we report a simple leaf-cutting transformation (LCT) method without aseptic operation using *Agrobacterium-*mediated genetic transformation with detached leaves to complete the transgenic operation. Jonquil (*Kalanchoe blossfeldiana*), an ornamental plant with leaf propagation ability, was selected to transform via the LCT method using *A. tumefaciens* strain EHA105 and *Agrobacterium rhizogenes* K599. Without selection pressure, the visual reporter *Ruby*, a system for the biosynthesis of betalains, was used in the LCT method. New colorful germplasms of Jonquil accumulated with betalains were obtained. The transgenic Jonquils transformed by *A. tumefaciens* EHA105 grow normally, but these transgenic Jonquils transformed by *A. rhizogenes* K599 have abnormal growth, manifested as dwarf phenotype, and most Jonquils had protrusions like tentacles or polyps on their leaves. Stable transformation in Jonquil should be mediated by *A. tumefaciens*, not *A. rhizogenes.*

## Introduction

*Agrobacterium* species are renowned for their role in creating genetically modified plants, thanks to their ability to integrate transfer DNA (T-DNA) into the nuclear chromosomes of plant hosts (Ziemienowicz, 2014). *Agrobacterium tumefaciens* and *Agrobacterium rhizogenes* are the most widely used *Agrobacterium* species in plant genetic engineering (Flores-Félix et al., 2020). The *A. tumefaciens* possesses a tumor-inducing (Ti) plasmid, which it employs to deliver its genetic material into the host’s chromosome. *A. tumefaciens-*mediated genetic transformation has become the predominant transformation approach in numerous plants. It is widely employed to introduce foreign genes into plants to study gene function or create new germplasm (Hiei et al., 2014; Yadava et al., 2017; Yu et al., 2024). The CRISPR/Cas9 editing technology has emerged as a powerful tool in gene function research and the development of novel plant germplasm (Gao, 2021). In the CRISPR/Cas9 gene editing process, the genetic transformation process is a critical step that involves effectively delivering the CRISPR/Cas9 system (including Cas9 protein and guide RNA) to target cells. *A. rhizogenes* contains a root-inducing (Ri) plasmid, and a cluster of rooting locus (*rol*) genes in the T-DNA of Ri can lead to produce hairy roots in recipient plants (Rugini et al., 1991; Bulgakov, 2008). Therefore, *A. rhizogenes* is often used to induce plants to produce hairy roots and form composite plants, becoming a powerful tool for studying rhizobium legume symbiosis, plant root systems, and other research.

In the process of plant genetic transformation, some plants need to induce callus first. For example, monocotyledonous plants such as rice, corn, and wheat induce callus through mature embryos or young embryos, and then infect the callus through *A. tumefaciens-*mediated transformation or particle bombardment. The infected callus is further induced to differentiate into buds, then the roots were produced at the base of the stem (Kausch et al., 2021; Wang et al., 2022). Many dicotyledonous plants, such as plants of legumes and Solanaceae, are directly infected with *A. tumefaciens* through leaves, cotyledons, or hypocotyls as explants. The infected explants are placed on the differentiation medium for callus induction and bud differentiation (Choudhury and Rajam, 2021). The whole transformation process using the *A. tumefaciens-*mediated genetic transformation method based on tissue culture is carried out in a sterile environment. For most crops, regeneration and genetic transformation are still arduous and highly dependent on species and genotype (Shin et al., 2020; Kausch et al., 2021; Maren et al., 2022; Wang et al., 2022). Some regulatory genes related to growth and development have been identified. Remarkably, these genes can notably enhance regeneration efficiency, curtail the transformation time, and even enable the transformation of recalcitrant genotypes (Maren et al., 2022; Wang et al., 2022; Yang et al., 2024). These studies still focused on how to improve the transformation efficiency and break through the restriction of genotype or species, but they do not reduce the process and steps of genetic transformation operation.

Traditional *A. tumefaciens-*mediated genetic transformation requires tissue culture in a sterile environment, which is cumbersome and time-consuming, and has high requirements in terms of professional skills and experience. Consequently, streamlining this approach has been regarded as a valuable objective. Moreover, achieving plant transformation without the need for tissue culture is extremely appealing. Floral dip is typically employed for genetic transformation in *Arabidopsis thaliana*, featuring a simple operation that does not require sterile procedures or tissue culture, aiding in its establishment as a model plant. Regeneration-dependent strategies for new plants can be carried out either within a living plant (*in planta*) or outside of a living plant (*ex planta*) (Bélanger et al., 2024), such as the Fast-TrACC (fast-treated agrobacterium co-culture) method that was developed in *Nicotiana benthamiana* and tomato (Maher et al., 2020). A simple and effective *A. tumefaciens*-mediated transformation method named regenerative activity-dependent *in planta* injection delivery (RAPID) was established in sweet potato. The RAPID method does not require tissue culture, overcoming the technical limitations of existing transformation strategies (Mei et al., 2024).

In nature, numerous plants possess the characteristic of root tillering, They are capable of spontaneously sprouting from their roots and subsequently developing stems and leaves. The cut–dip–budding (CDB) method was developed using *A. rhizogenes*-mediated genetic transformation in root-suckering plants (Cao et al., 2023). Similarly, the CDB delivery system has been constructed with *A. rhizogenes*-mediated genetic transformation and gene editing in succulent plants (Lu et al., 2024). The CDB method did not require an aseptic operation, but it was mediated by *A. rhizogenes* K599. However, when *A. rhizogenes* was used for stable genetic transformation, the resulting transgenic plants often exhibited defects. For instance, transgenic sweet potatoes transformed from *A. rhizogenes* had wrinkled leaves, and smaller storage roots with many fibrous roots (Otani et al., 1993). The expression of *rolB2* gene derived from *A. rhizogenes* A4 led to a reduction in the growth of Jonquil (*Kalanchoe blossfeldiana*) plants and resulted in a more compact growth habit (Favero et al., 2021). Similarly, ornamental plants like *Sinningia* sp*eciosa* transformed with *A. rhizogenes* exhibited shortened peduncles, wrinkled leaves, and delayed flowering (Vereshchagina et al., 2023). The regenerated plants obtained with *A. rhizogenes* K599 showed wrinkled leaves and more branches when compared to the wild-type Chinese cabbage (*Brassica rapa*) (Wang et al., 2024). However, the CDB method did not specify whether the transgenic root-suckering plants derived from *A. rhizogenes* K599 grew normally.

To investigate whether genetic transformation using *A. rhizogenes* K599 in the ornamental plant Jonquil, which has a strong leaf propagation ability, would lead to abnormal growth, we employed a simple leaf-cutting transformation (LCT) method to transform it using *A. rhizogenes* K599 and *A. tumefaciens* EHA105. The LCT method does not require the aseptic operation to complete the transgenic operation. The visual *Ruby* reporter, which is composed of three key genes (*CYP76AD1*, *DODA*, and *glucosyltransferas*e) involved in betalain biosynthesis, was used to monitor transgenic events in this study (He et al., 2020).

## Results

### Transgenic Jonquil produced with *A. tumefaciens* EHA105 grows normally

**Figure 1:**
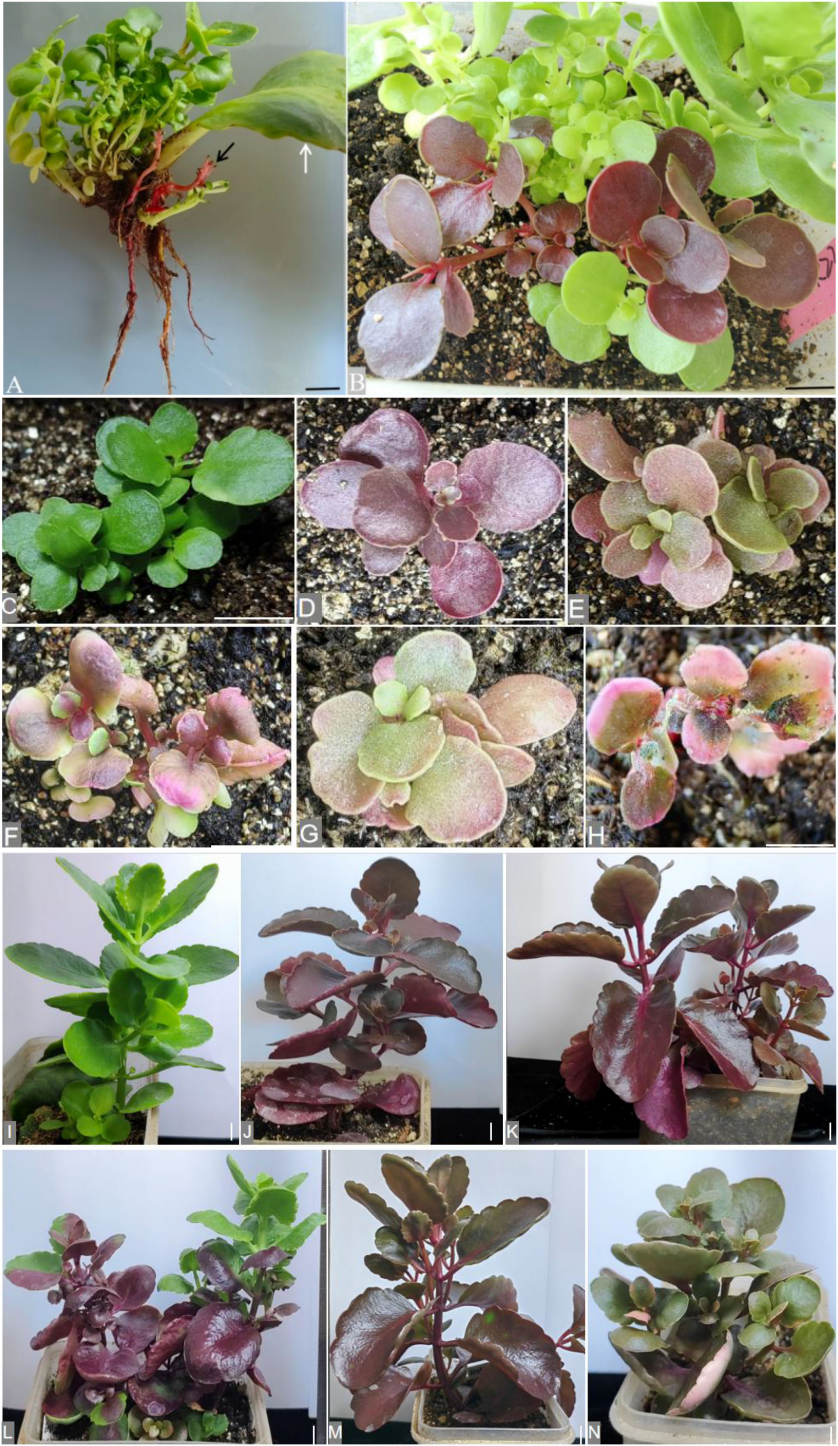
The transgenic Jonquil produced via the LCT method using *A tumefaciens* strain EHA105. **(A)** Buds formed at the infected part of the petiole of Jonquil after 12 weeks of transformation. Black arrow: new red buds grow from the propagated leaves. White arrow: propagative leaf. **(B)** After approximately 15 weeks of transformation, the seedlings of Jonquil appeared with vividly red color. **(C–H)** One-week-old plants after transplant; **(I–N)** 8-week-old plants after transplant; **(C, I)** regenerated wild type. **(D–H, J–N)** Independent lines with red color. Bars = 1 cm.

After infecting the propagated leaves with *Agrobacterium* for 12 weeks of culturing in vermiculite, the cluster of buds formed at the infected part of the petiole of Jonquil (**Figure 1A**). All explants produced buds, and the regeneration efficiency in Jonquil leaves was 100%, indicating that the Jonquil leaves have a strong reproductive ability. A total of 16 independent explants produced red buds with red roots (**Figure 1A**) and also green buds. After 15 weeks, these buds developed into Jonquil seedlings (**Figure 1B**) and then were transplanted into soil (**Figures 1C–H**). The leaves of red-colored Jonquil seedlings exhibited a variety of red color (**Figures 1D–H**).

**Figure 2:**
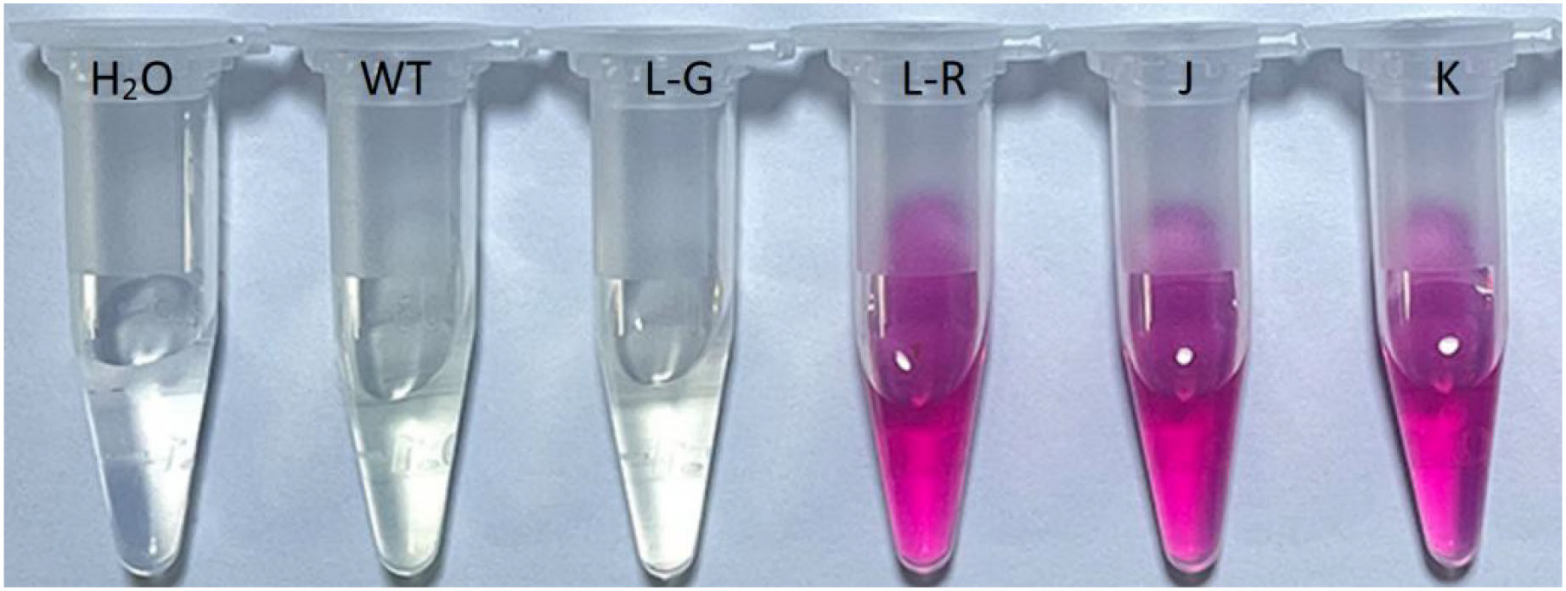
Betalain extract from leaves of red-colored Jonquil plants. H_2_O: water, WT: regenerated wild type, L-G: from the green part of one leaf showed red and green color in the independent line L in **Figure 1**, L-R: from the red part of one leaf showed red and green color in the independent line L in **Figure 1**, J: the independent line J in **Figure 1**, K: the independent line K in **Figure 1**.

After 8 weeks of transplantation, the red-colored Jonquil grew normally like the green plants (**Figures 1I–N**). The height of the red-colored Jonquil plant was approximately 18 cm (**Table 1**). Interestingly, there are both red- and green-colored leaves in one red-colored line, and even one leaf showed red and green colors (**Figure 1L**). The betalain measurement showed that the green part of the color mosaic leaf did not contain betalain, while the red part contained betalain in the color mosaic plant (line L in **Figure 1**) (**Figure 2**).

**Table 1:** Plant height, leaf size, and content of betalain of transgenic jonquil plants.

Transformation lines	Plant height (cm)	Leaf size (length/width) (cm)	Content of betalain (mg/g, fresh weight)
Wild type of Jonquil	16.7	4.5/3.4	0.000
Line J from EHA105	18.2	5.1/4.0	0.162
Line K from EHA105	17.6	4.5/3.4	0.169
Line L from EHA105	18.4	5.2/4.2	0.170
Line M from EHA105	16.5	4.6/3.5	0.151
Line N from EHA105	18.3	4.8/3.6	0.103
Line 1 from K599	4.2	2.5/2.6	0.111
Line 2 from K599	4.6	2.2/2.6	0.086
Line 3 from K599	3.5	1.5/1.7	0.071
Line 4 from K599	4.3	2.5/2.0	0.041
Line 5 from K599	4.4	1.8/2.1	0.121

### Transgenic Jonquil produced with *A. rhizogenes* K599 grows slowly

**Figure 3:**
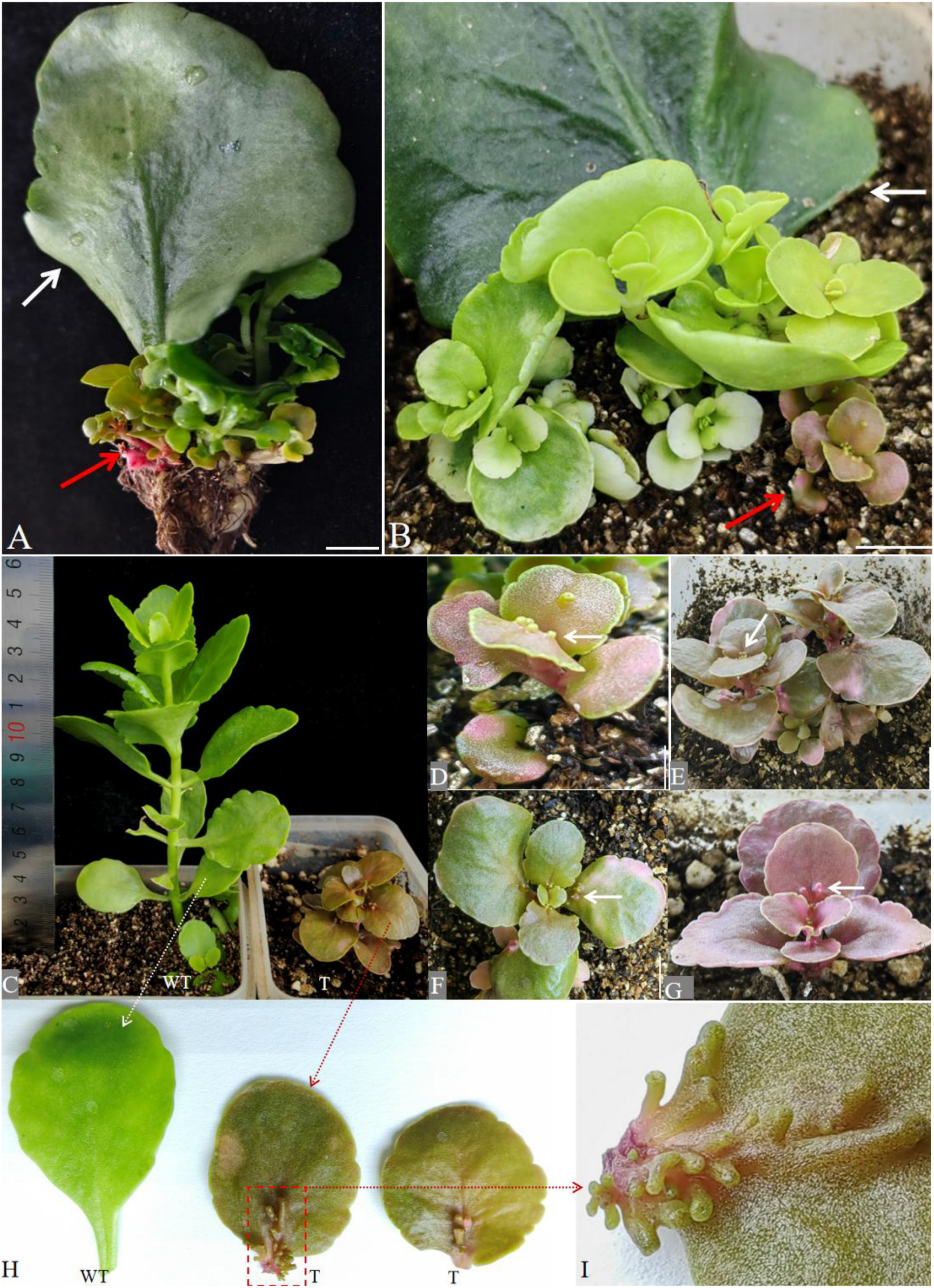
The transgenic Jonquil produced via the LCT method using *A rhizogenes* K599. **(A)** Buds and **(B)** seedlings formed at the infected part of the petiole of Jonquil after 12 and 15 weeks of transformation. Red arrows: new red buds grow from the propagated leaf. White arrows: propagative leaf. **(C)** Regenerated wild-type (WT) and transgenic (T) Jonquil. **(C–G)** Independent lines with red-colored Jonquils after 8 weeks of transplantation. White arrows: protrusions such as tentacles or polyps. **(H)** Leaves of regenerated Jonquil. WT: leaf of wild-type Jonquil; T: leaves of red-colored Jonquil. **(I)** Enlarged leaf part with protrusions such as tentacles or polyps. Bars = 1 cm.

New shoots were produced at the petiole incisions of all 60 explants of Jonquil after 12 weeks of culture (**Figure 3A**) and the regeneration efficiency of Jonquil leaves is 100%. Only six independent leaves have produced red color shoots with red roots from the petiole of Jonquil leaves (**Figures 3A, B**).

After 8 weeks of transplantation, the height of the wild-type Jonquil was already 15 cm, while all these red-colored Jonquil plants grew slowly and the height of red-colored Jonquil was only 3–4 cm (**Figures 3C–G**). Among the six red-colored lines obtained with A. rhizogenes K599, five lines had protrusions like tentacles or polyps on their leaves. The number of protrusion structures such as tentacles or polyps differs in different leaves (**Figures 3C–I**). The length and width of the leaves obtained from K599 transformation are significantly reduced compared with wild-type Jonquil. Three mature leaves in the middle and lower parts of the plant were measured, and the average values were used to evaluate the size of the leaves (**Table 1**).

### PCR identification of red-colored Jonquils

**Figure 4:**
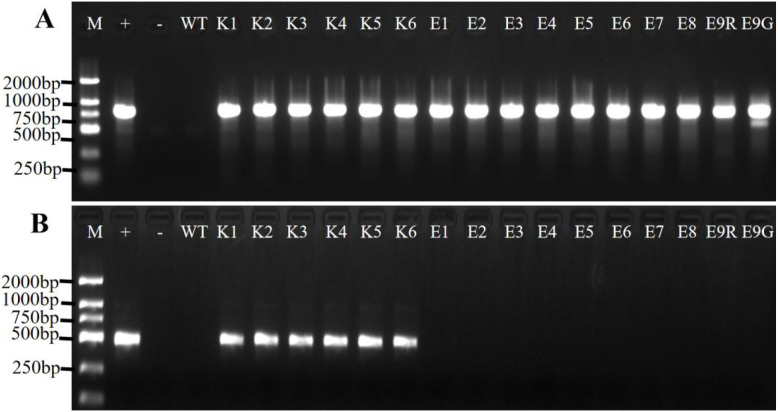
PCR validation with the transgenic Jonquil lines. **(A)** PCR validation of transformation lines with DoDF/R. **(B)** PCR validation of the *RolB* gene with the transgenic Jonquil lines. Lane M, DL2000 DNA marker; Lane +, p35RUBY plasmid; Lane -, negative control (water as template); WT, negative control (propagative leaf); K1–K6, independent transgenic Jonquil lines obtained from *A rhizogenes* K599; E1–E9, independent transgenic Jonquil lines obtained from *A tumefaciens* EHA105; E9R and E9G, red-colored and green-colored part form color chimera Jonquil, respectively.

All of the red-colored Jonquils underwent amplification, resulting in the acquisition of an 818-bp fragment. In contrast, when using the propagative leaf as a negative control, no amplified fragment was detected (**Figure 4A**). The results showed that these red-colored Jonquils were transgenic plants. The genetic transformation efficiencies of *A. tumefaciens* EHA105 and *A. rhizogenes* K599 were 16% and 10%, respectively (**Table 2**).

**Table 2:** Transformation efficiency with *Agrobacterium* using the LCT method.

*Agrobacterium*	Explants	Regeneration efficiency	Buds with red color	Transformation efficiency
*A. tumefaciens* HA105	100	100%	16	16%
*A. rhizogenes* K599	60	100%	6	10%

One chimera Jonquil (line E9) with both red- and green-colored leaves was found (**Figures 1F, L**). Genomic DNAs were extracted from the red and green parts of one leaf, separately. DODA gene was amplified from both parts (**Figure 4A**), indicating that the color chimera Jonquil was not a transgenic chimera, which may be caused by the silencing of the Ruby gene.

### PCR identification with RolB gene on the transgenic Jonquil plants produced with *A. rhizogenes* K599

The PCR amplification results showed that the 482-bp DNA fragment of *RolB* gene can be amplified from all the red-colored Jonquils obtained from *A. rhizogenes* K599, while there is no amplification product in the red-colored Jonquils obtained from *A. tumefaciens* EHA105 (**Figure 4B**). This indicated that these red-colored Jonquils obtained from *A. rhizogenes* K599 carried the *RolB* gene, which may have been introduced during the genetic transformation process mediated by *A. rhizogenes* K599.

## Discussion

In nature, some plants can propagate by *ex vivo* leaves; that is to say, the *ex vivo* leaves of these plants can produce buds without exogenous hormones added. In this study, the regeneration efficiency of the *ex vivo* leaves of Jonquil is 100%; thus, Jonquil has a strong leaf propagation ability. The wild*-*type *A. tumefaciens* contains a Ti plasmid, and the T-DNA of Ti plasmid contains genes that can be integrated into the plant genome (Barton et al., 1983). These genes in T-DNA of the Ti plasmid can change the metabolic pathways of plant cells to produce phytohormones (such as auxin and cytokinin) and some amino acid derivatives, thereby promoting cell proliferation and forming crown galls (Baron and Zambryski, 1996). *A. tumefaciens* currently employed in plant genetic transformation has undergone artificial modification. Specifically, those genes within the T-DNA that are responsible for inducing crown galls have been deleted. By connecting the target gene into the plant binary vector and then transforming it into the modified *A. tumefaciens*, the target gene can be introduced into plant cells and stable inheritance can be achieved by infecting the explants with *A. tumefaciens* (Hoekema et al., 1983). Wild*-*type *A. rhizogenes* contains Ri plasmid and conveys its Ri plasmid to the host plant during infection and triggers the growth of a root mass characterized by excessive root proliferation at the infection site (Loyola-Vargas et al., 2024). The *rol* gene group on the T-DNA of Ri plasmid leads to the production of hairy roots by changing the hormone balance or the sensitivity of cells to auxin and cytokinin. These hairy roots have the characteristics of rapid growth (Rugini et al., 1991; Bulgakov, 2008). *A. rhizogenes* K599 contains pRi2659 and can induce hairy roots in transformation-recalcitrant plants, like soybean, and has higher transformation efficiency than other strains (Valdes Franco et al., 2016). Transgenic sweet potatoes were obtained with *A. rhizogenes*-mediated transformation, and the leaves developed from transgenic root tubers were wrinkled, the flower shape was changed, the apical dominance decreased, the internodes shortened, and the storage roots became smaller and grew a lot of fibrous roots; these plants had frequent branches and reduced geotropism (Otani et al., 1993). In this study, all these red-colored Jonquil plants grew slowly, and more than half of transgenic Jonquil plants had protrusions like tentacles or polyps on their leaves. The *RolB* gene was also amplified from all the red-colored Jonquils obtained from *A. rhizogenes* K599. Combined with the research results of *A. rhizogenes*-mediated genetic transformation in Jonquil, *S.* sp*eciosa*, and Chinese cabbage, the transgenic plants showed leaf curling and compact plant architecture (Favero et al., 2021; Vereshchagina et al., 2023; Wang et al., 2024). Therefore, we believe that the phenomena of dwarf symptoms and abnormal leaves are caused by the integration of the plasmid Ri of *A. rhizogenes* K599 into plants as well. Therefore, *A. tumefaciens* should be used in plant stable genetic transformation, while *A. rhizogenes* was usually used to produce composite plants.

There are numerous reports on the method of using hypocotyl injection to induce hairy roots (Kereszt et al., 2007; Meng et al., 2019). After the appearance of hairy roots at the injection site of the stem, the hairy roots are directly exposed to the air and do not come into contact with soil or other media. Therefore, when producing hairy roots by injecting hypocotyls, it is impossible to add antibiotics or herbicides to kill *Agrobacterium* or select transgenic positive roots. To prevent *Agrobacterium* from becoming an endophyte of the plant during *Agrobacterium*-mediated genetic transformation through propagative leaf, we recommend incorporating a cefotaxime cleaning phase. This step should be carried out 3 days after the plant has been infected with *Agrobacterium*. Nevertheless, we do not advocate the addition of selective antibiotics or herbicides. The underlying reason is that the process of shoot regeneration achieved through the propagation of leaves is highly dependent on the regulation of endogenous hormones within the propagated leaves. This endogenous hormone-mediated regulation plays a crucial role in triggering and facilitating the generation of regenerated shoots, and the introduction of selective antibiotics or herbicides may disrupt this delicate hormonal balance, potentially impeding the successful regeneration of shoots. There is no selection pressure using the LCT method, so the visual reporter gene is preferred. The visualized reporter gene can easily distinguish transgenic plants from non-transgenic plants. The genes that are frequently employed and can function as visualized reporter genes encompass β-glucuronidase (GUS), luciferin, fluorescent protein gene, anthocyanin synthesis regulatory factor (such as *AtMYB75*), and *Ruby* (He et al., 2020; Lyu et al., 2024). GUS staining assay is destructive to plant tissues and requires expensive chemical substrates (X-Gluc). Luciferin and fluorescent proteins stand out because of their ability to serve as a non-destructive, visual indicator, but they require optical equipment and/or substrates. Anthocyanin synthesis regulators are limited by the genotype of plant varieties. The reporter system *Ruby* overcomes the constraints of chemical substrates and optical instruments and can be used in all plant cells (He et al., 2020). *Ruby* is widely used as a reporter gene in many plants (Wang et al., 2023; Yu et al., 2023; Lyu et al., 2024). The reporter *Ruby* can also realize quantitative evaluation of gene expression level and promoter activity (Liu et al., 2024). *Ruby* was used to develop a splicing reporter, enabling the direct visual observation of pre-mRNA splicing (Prasad et al., 2024).

In this study, the genetic transformation efficiency is 16% and 10% with *A. tumefaciens* EHA105 and *A. rhizogenes* K599, respectively, with the LCT method using the *Ruby* reporter gene without selection pressure, while the transformation efficiency mediated by *A. rhizogenes* K599 was 74% with the CDB delivery system (Lu et al., 2024). The genotypes of the materials and infection solution used in these two methods are different, which may be the main reason for the difference in transformation efficiency. Using the CBD method, leaf segments used as explants were immersed in *A. rhizogenes* K599 infection solution [10 mM MgCl2, 10 mM 2-(N-morpholino) ethanesulfonic acid (MES), and 100 μM acetosyringone (AS), pH 6.0]. In this study, leaf segments were soaked with bacterial infection solution (OD_600_ = 0.6) (4.43 g/L M519 + 30 g/L sucrose + 2 mg/L 6-BA + 10 μmol/L AS; pH 5.8). There are differences in the infection solution used for these two transformation methods. MES and 100 μM AS were added in the CBD method, while only 10 μmol/L AS was used in the LCT method. Perhaps due to the absence of MES and the use of lower concentrations of AS, the transformation efficiency of the LCT method decreased. The genetic transformation efficiency of soybean is very low, and adding MES in the medium of genetic transformation in soybean can improve the transformation efficiency (Olhoft et al., 2003). In this study, an area of 32 cm × 45 cm in the culture room was occupied to obtain more than 10 independent transgenic lines; thus, the LCT method is suitable for high-throughput transgenic operations. The LCT method is very simple and does not require aseptic operation. There is no need to change the culture medium containing different hormones as required by the traditional transformation methods. The LCT method can be applied to the transgenic modification of plants that propagate asexually through leaves.

## Conclusion

In summary, we have constructed a simple LCT method using *Agrobacterium*-mediated genetic transformation with detached leaves without aseptic operation to complete the transgenic operation. The LCT method is suitable for plants with leaves that have reproductive ability. The transgenic Jonquils transformed by *A. tumefaciens* EHA105 grew normally, but these transgenic Jonquils transformed by *A. rhizogenes* K599 had abnormal growth, manifested as a dwarf phenotype, and most Jonquils had protrusions like tentacles or polyps on their leaves. Consequently, stable transformation in Jonquil should be mediated by *A. tumefaciens*, not *A. rhizogenes*.

## Methods

### Plant materials and growth conditions

The Jonquil variety “Baishuijing” was purchased from the local flower market. These Jonquils were 2 years old and cultivated in a growth chamber (24–26°C, 16-h light/8-h dark cycle) with light intensity at 300 µmol·m^−2^·s^−1^. MS power medium (M519) was purchased from Phyto Technology laboratories (Shawnee Mission, Kansas).

### *Agrobacterium-*mediated transformation

p35RUBY was constructed in our previous study (Lyu et al., 2024) and was transformed into *A. tumefaciens* strain EHA105 and *A. rhizogenes* K599 using electroporation. EHA105 and K599 harboring with p35RUBY were cultured in LB medium with 50 mg/L kanamycin and 50 mg/L rifampicin, and 50 mg/L kanamycin and 50 mg/L streptomycin, respectively, at 28°C. One clone was picked from *Agrobacterium* harboring with p35RUBY and cultured in liquid LB medium with the corresponding antibiotics. Bacterials were collected by centrifugation with 2,200*g* for 10 min at room temperature and resuspended with bacterial suspension solution (OD_600_ = 0.6) (4.43 g/L M519 + 30 g/L sucrose + 2 mg/L 6-BA + 10 μmol/L AS; pH 5.8).

The LCT method is the same as our previous one-step ARM hair root transformation (Fan et al., 2020). One- to two-month-old leaves as explants were cut from adult Jonquil plants. The incision of the petiole was soaked in the bacterial suspension solution of *Agrobacterium* harboring p35RUBY for 30 min (**Supplementary Figure 1A**) and then coated with the *Agrobacterium* bacterial harboring p35RUBY (**Supplementary Figure 1B**). The leaves were cultivated in sterile vermiculite wetted with sterile 1/10 MS solution (0.43 g/L M519) (**Supplementary Figure 1C**). All the explants were covered with transparent plastic bags to maintain humidity (**Supplementary Figure 1D**). Water was added every 10 days to maintain the humidity of vermiculite. There are 100 and 60 leaves used for the transformation of *A. tumefaciens* strain EHA105 and *A. rhizogenes* K599, respectively.

### PCR identification with *RUBY*

To characterize the transgenic lines, the *DODA* gene, one gene of *Ruby*, was utilized for identifying the transgene events. The sequence of the *DODA* gene can be found in He et al. (2020). One leaf of independent line was subjected to DNA isolation. Genomic DNA was extracted according to a previously described method (Fan et al., 2020). The forward primer DoDF (5′-GATGAACGGCGAGGACG-3′) and the reverse primer DoDR (5′-CGGCGGAGGTGAACTTG-3′) were used for the PCR analysis. The PCR program was performed as follows: 94°C for 3 min, then 94°C for 30 s, 58°C for 30 s, and 72°C for 40 s, with 35 cycles, and finally 72°C for 5 min.

The fragment amplified with the primer pair DoDF/DoDR is expected to be 818 bp in size. All red-colored Jonquils were amplified with an expected 818-bp fragment, with the wild-type leaf of Jonquil as a negative control and p35RUBY plasmid as the positive control.

### *RolB* gene identification with PCR amplification

*A. rhizogenes* K599 carries the pRi2659 plasmid. The sequencing of pRi2659 plasmid has been completed and the sequence was released in GenBank No. CP019703 (Mankin et al., 2007). The pRi2659 plasmid contains a cluster of *rol* genes in the T-DNA. *RolA*, *RolB*, *RolC*, and *RolD* in the cluster of *rol* genes are involved in auxin/cytokinin imbalance (Mankin et al., 2007; Kyndt et al., 2015). The primer set of the *RolB* gene, the forward primer rolB3 (5′-AGGGCGAGGTCGCTACGA-3′) and the reverse primer rolB4 (5′-GGCGGCGTACTTTAAATGGC-3′), was designed based on plasmid pRi2659, and the PCR fragment is expected to be 482 bp in size. PCR amplification was performed with the genomic DNA extracted from the red-colored Jonquil plants. The PCR program was performed as follows: 94°C for 3 min, then 94°C for 30 s, 63°C for 30 s, and 72°C for 30 s, with 35 cycles, and finally 72°C for 5 min.

### Betalain pigment measurement

Betalains were extracted based on the reported method with modification (Chen et al., 2023). A total of 0.5 g of fresh leaf was cut from Jonquil plants and then was immersed into 50 mL of 100% ethanol to remove chlorophyll pigments for 18 h at room temperature. The leaf was then transferred into 5 mL of water for 12 h at room temperature. The 5 mL extraction mixtures were used to measure. The light absorbance of the extraction mixtures was measured in a spectrophotometer at 538 nm wavelength. The calculation of betalain concentrations was detailed in the reported research (García-Cruz et al., 2013). Three biological replicates were performed for each independent line.

## Data Availability

The original contributions presented in the study are included in the article/**Supplementary Material**. Further inquiries can be directed to the corresponding author.
