# Supplementary material for: Stable transformation mediated by Agrobacterium tumefaciens in Jonquil is better than Agrobacterium rhizogenes
Source: Front Plant Sci. 2025 Jun 26;16:1594197. doi: 10.3389/fpls.2025.1594197 (PMC12243028; doi:10.3389/fpls.2025.1594197)
Supplement: Supplementary file 1 [file Table1.docx]

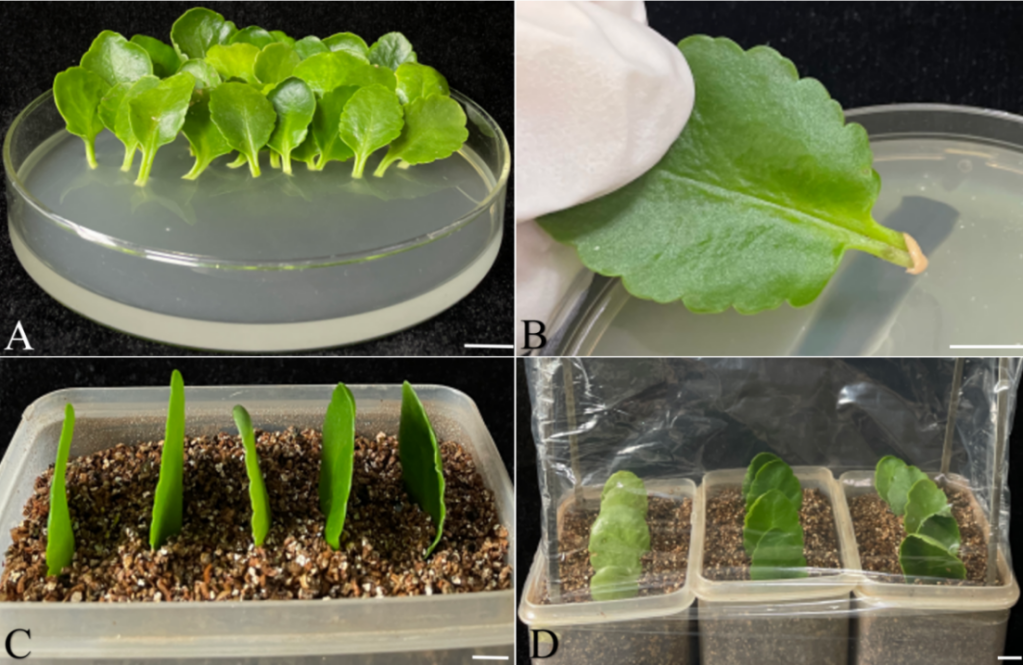


**Supplementary Figure 1*.*** The LCT method process with Agrobacterium using 1-2 months old leaves of Jonquil as explants. (A) The petioles of leaves was soaked in the bacterial suspension. (B) The petiole incision was coated with bacteria. (C) The leaves were cultivated in sterile vermiculite. (D) All the explants were covered with a high light transparent plastic bags to maintain humidity. Bars=1cm.
